# Mapping the complexity of care in patients with heart disease living with chronic kidney disease

**DOI:** 10.1093/ckj/sfag242

**Published:** 2026-08-07

**Authors:** Kaile Chen, Hong Xu, Farhad Abtahi, Antoine Creon, Carlos Fernandez-Llatas, Fernando Seoane, Juan-Jesus Carrero

**Affiliations:** Department of Clinical Science, Intervention and Technology, Karolinska Institutet, Stockholm, Sweden; Department of Biomedical Engineering and Health Systems, School of Engineering Sciences in Chemistry, Biotechnology and Health, KTH Royal Institute of Technology, Huddinge, Sweden; Department of Medical Epidemiology and Biostatistics, Karolinska Institutet, Stockholm, Sweden; Department of Medical Epidemiology and Biostatistics, Karolinska Institutet, Stockholm, Sweden; Division of Clinical Geriatrics, Department of Neurobiology, Care Sciences and Society (NVS), Karolinska Institutet, Stockholm, Sweden; Department of Clinical Science, Intervention and Technology, Karolinska Institutet, Stockholm, Sweden; Department of Biomedical Engineering and Health Systems, School of Engineering Sciences in Chemistry, Biotechnology and Health, KTH Royal Institute of Technology, Huddinge, Sweden; Department of Clinical Physiology, Karolinska University Hospital, Stockholm, Sweden; Department of Medical Epidemiology and Biostatistics, Karolinska Institutet, Stockholm, Sweden; Department of Clinical Science, Intervention and Technology, Karolinska Institutet, Stockholm, Sweden; Institute of Information and Communication Technologies (SABIEN-ITACA), Universitat Politècnica de València, Camino de Vera S/N, Valencia, Spain; Department of Clinical Science, Intervention and Technology, Karolinska Institutet, Stockholm, Sweden; Department of Clinical Physiology, Karolinska University Hospital, Stockholm, Sweden; Department of Medical Technology, Karolinska University Hospital, Stockholm, Sweden; Department of Medical Epidemiology and Biostatistics, Karolinska Institutet, Stockholm, Sweden; Division of Nephrology, Department of Clinical Sciences, Karolinska Institutet, Danderyd Hospital, Stockholm, Sweden

**Keywords:** care complexity, chronic kidney disease, disease trajectories, heart failure, myocardial infarction, process mining

## Abstract

**Introduction:**

Patients with myocardial infarction (MI) or heart failure (HF) often have concomitant chronic kidney disease (CKD). These patients are perceived as ‘complex’, but empirical evidence quantifying this complexity or exploring the diseases and problems they experience is scarce. We aimed here to comprehensively characterize the complexity of care among patients with MI/HF across the spectrum of CKD severity and to identify frequent longitudinal disease accrual trajectories associated with all-cause mortality.

**Methods:**

Retrospective cohort of 29 901 adults surviving an incident MI/HF hospitalization from the Stockholm CREAtinine Measurements (SCREAM, 2006–2021) project. KDIGO CKD stages were classified as no CKD, mild CKD, or moderate-to-severe CKD using plasma creatinine and urinary albumin at hospital admission. Care complexity was evaluated through multidimensional indicators encompassing comorbid conditions, treatments, healthcare use, and mortality. Process mining identified longitudinal trajectories of disease accrual and associated mortality risks.

**Results:**

Increasing CKD severity was associated with greater comorbidities, polypharmacy, healthcare use, and higher observed rate of recurrent MI/HF and death. We identified 8927 hospitalizations and 20 805 outpatient disease trajectories during follow-up. CKD patients more often accumulated multiple circulatory diagnoses, significantly increasing mortality hazards compared with those without CKD. Trajectories involving progression to endocrine/metabolic, respiratory, neoplastic, and genitourinary diseases became more frequent with advancing CKD and were strongly associated with death.

**Conclusions:**

Among MI/HF survivors, those with CKD are the most complex and resource-demanding. We identified key comorbidity patterns that provide opportunities for earlier, targeted interventions—particularly addressing endocrine, respiratory, and neoplastic complications—to reduce their adverse outcomes and premature mortality.

KEY LEARNING POINTS **What was known:**

- Patients with myocardial infarction (MI) or heart failure (HF) frequently have coexisting chronic kidney disease (CKD), a combination perceived as clinically complex, but often lacking objective quantification of such complexity.
- Patients with MI–HF and CKD are at higher risk of adverse clinical outcomes. Studies evaluate one outcome at a time, but it is possible that disease occurrence clusters in trajectories.

**This study adds:**

- This study provides objective quantification of the higher complexity of care required by MI/HF survivors with CKD, demonstrating significantly greater multimorbidity, polypharmacy, and healthcare utilization across all levels of care with more severe CKD.
- This study also identified specific disease trajectories that more often cluster in people with CKD and that are associated with the risk of premature death: patients with CKD were more likely to develop endocrine, respiratory, neoplastic, and genitourinary diseases.

**Potential impact:**

- These findings highlight opportunities for early, multidisciplinary interventions to prevent these disease pathways.

## INTRODUCTION

Patients with myocardial infarction (MI) or heart failure (HF), collectively referred to as cardiovascular disease (CVD) [1, 2], frequently have coexisting chronic kidney disease (CKD) [3]. The coexistence of CVD and CKD is common because these conditions share major risk factors—including hypertension, diabetes, and vascular ageing—and accelerate each other’s onset [4].

In clinical practice, patients with both CVD and CKD are often perceived as ‘complex.’ This perception stems from their high burden of comorbidities [5], which complicates therapeutic decision-making and follow-up. In addition, cardiovascular treatment strategies often differ in this population, in part because patients with CKD have historically been excluded from major clinical trials, leading to concerns about treatment efficacy and safety [6, 7]. Providing empirical evidence on this perceived complexity is important to set up the stage for the upcoming *European Society of Cardiology* clinical guideline on the management of CKD in patients with CVD, expected to be released in late 2026 [8]. In addition, while there is a broad consensus that complex patients require more time and resources to achieve high-quality care, reimbursement models for hospitals and physicians are typically structured around patient numbers rather than the intensity of care required [9]. Furthermore, clinic schedules generally allocate time according to patient volume, without explicit consideration of clinical complexity [9].

Over the last two decades, advances in evidence-based treatments and procedures have substantially improved survival after CVD. However, this progress has also resulted in an expanding population of survivors who remain vulnerable to adverse outcomes, including hospital readmissions, new chronic diseases, reduced quality of life, higher healthcare costs, and premature mortality [10, 11]. Although studies in secondary CVD prevention have established associations between low kidney function and individual outcomes [12], no study has comprehensively described the sequential accrual of diseases among these patients. Understanding how diseases accumulate over time is a prerequisite for identifying points where early intervention could interrupt or delay the most detrimental pathways.

To address this gap, we analysed rich routine care data from a large Swedish health system with the primary aim of comprehensively quantifying the complexity of care in patients with MI/HF across the presence and severity of CKD. We evaluated this complexity through a multifaceted lens, including the prevalence of multimorbidity and polypharmacy, the frequency of healthcare interactions, and the risk of adverse clinical outcomes. As a secondary aim, we then used process mining, a data-driven analytic method designed to identify and visualize sequential patterns in clinical events [13], to characterize common disease trajectories experienced by these patients and that precede death. Such knowledge may help clinicians, patients, and caregivers anticipate the likely course of illness and support the design of more appropriate care pathways.

## MATERIALS AND METHODS

### Data source and study design

We used data from the Stockholm CREAtinine Measurements (SCREAM) healthcare-utilization cohort [14], a population-based cohort that captures all residents in the Stockholm region. SCREAM includes laboratory data and is linked with regional and national registers that hold complete information on health-care contacts, dispensed prescriptions, renal replacement therapy, death, and migration data. We conducted a cohort study design to capture post-MI/HF trajectories of diagnoses, medication use, and healthcare visits occurring between 1st January 2006 and 31st December 2021. This study complied with the Declaration of Helsinki and was approved by the Swedish National Board of Welfare and the Stockholm Regional Ethics Review Board (Ethical Approval nr. 2017/793-31).

### Study population

Adults aged 18 or older were included if they survived to discharge after an incident hospital admission for MI or HF (qualifying definitions listed in Table S1). To ensure that only incident MI/HF events were included, we excluded participants with a history of MI or HF as reflected through issued diagnosis in their medical records since 1997, the year when the ICD-10 [10th revision of the International Classification of Diseases (ICD-10)] system was implemented in Sweden. Next, to enable precise assessment of kidney disease (and severity), we excluded people who lacked any measurement of creatinine or albuminuria within the 18 months before the incident MI/HF hospital admission. Finally, we excluded individuals who died during the index hospitalization or were not residing in our region. The discharge date served as the index date from which follow-up began.

### Study exposure

The study exposure is the presence (and severity) of CKD, following the KDIGO classification [15] that combines measures of estimated glomerular filtration rate (eGFR) and albuminuria. eGFR was calculated with the revised Lund–Malmö equation [16] using creatinine, age, and sex. This equation is automatically reported in Swedish care and is used to guide clinical decisions. In addition, this eGFR equation shows the best approximation to measured GFR in Swedish patients [16]. For creatinine, we selected only outpatient tests, in either plasma or serum. For albuminuria, we extracted information on outpatient tests regardless of method: dipstick albuminuria or proteinuria tests, 24-h and spot albumin concentrations, urinary protein-to-creatinine ratio, and urinary protein or albumin-to-creatinine ratio (UACR). Urinary protein-to-creatinine ratio and dipstick tests were approximated to UACR values using previously validated equations [17]. Their concentrations were then categorized per KDIGO [15] as: A1, normal to mildly increased albuminuria, i.e. <30 mg/g; A2, moderately increased albuminuria, that is, 30–300 mg/g; and A3, severely increased albuminuria, that is, >300 mg/g. In cases with more than one eligible albuminuria or creatinine test in the 18 months prior to the index date, we selected the one closest in time.

Combining eGFR and albuminuria levels, we classified patients into 3 categories: **No-CKD** if both their eGFR and albuminuria were normal, i.e. eGFR was ≥90 ml/min/1.73 m² and normal to mildly increased albuminuria; CKD with stages 1–2 (from now on **CKD1–2**), if eGFR was 60–89 ml/min/1.73 m² with moderate to severely increased albuminuria; and CKD with stages 3–5 (from now on **CKD3–5**), if eGFR <60 ml/min/1.73 m² regardless of albuminuria levels. The latter category also included patients on maintenance dialysis and with a history of kidney transplantation, both of which were ascertained by linkage with the nationwide Swedish Renal Registry. A detailed definition of this classification is provided in Supplemental Table S1.

### Indicators of care complexity

We defined patient complexity through four multidimensional proxies: we first quantified the burden of illness through the number of identified comorbid conditions and ongoing treatments. Chronic comorbid conditions were those listed in a published framework [18] of 28 validated algorithms based on issued diagnoses (ICD-10th revision, detailed in Supplemental Table S2). Ongoing treatments were defined by the number of unique medications dispensed at any Swedish pharmacy in the 6 months prior to hospital admission, identified through distinct ATC codes as registered in the nationwide Swedish Medication Registry. Polypharmacy is commonly defined as the concurrent use of ≥5 medications, while ≥10 medications is used to define excessive polypharmacy [19, 20]. The second set of indicators evaluated short-term care complexity during the first year after the index MI/HF event: count of distinct diagnoses issued during their healthcare contacts and of unique dispensed medications. The third set of indicators of complexity quantified healthcare resource utilisation [21] during the entire follow-up, and defined their annual rate of hospital admissions, the number of days spent in hospital per year, the rate of outpatient visits to specialized care, and the rate of visits to primary care. The final care complexity outcome pertained to rates of HF/MI readmission and all-cause mortality.

We summarized these indicators as proportions with 95% confidence intervals, mean (standard deviation-SD), or median (Q1–Q3), depending on covariate distribution. For healthcare utilization, crude incidence rates were calculated as events/person-time and reported per 100 person-years; 95% confidence intervals were obtained using exact Poisson methods (poisson.test). We do not emphasize baseline hypothesis-testing *P*-values between CKD strata because small clinically negligible differences may become statistically significant in large cohorts.

### Longitudinal disease trajectories and associations with mortality

Disease trajectories were derived for each individual using the process mining framework [13]. Hospitalization episodes and outpatient specialist consultations were separately and chronologically ordered based on their start dates, enabling inference of disease accrual patterns from timestamped diagnostic events. Using Direct-Follows Graphs (DFG), disease accrual sequences were summarized in process maps. A ‘trajectory’ was defined as a distinct, complete sequence of events; patients sharing the same sequence were grouped into the same variant. A trajectory (variant) was therefore operationalized as a unique, ordered ICD-10 chapter-level sequence; patients with identical sequences were assigned to the same variant. Disease trajectories were based on the primary and secondary diagnoses recorded at each visit in healthcare encounter during follow-up, using the ICD-10 codes grouped at the chapter level of the following diseases: A00–B99, infectious/Parasitic diseases; C00–D48, neoplasms; D50–D89, diseases of the blood and blood-forming organs; E00–E90, endocrine, nutritional and metabolic diseases; F00–F99, mental and behavioural disorders; G00–G99, diseases of the nervous system; H00–H59, eye and adnexa; H60–H95, ear and mastoid process; I00–I99, circulatory system; J00–J99, respiratory diseases; K00–K93, digestive system; L00–L99, skin and subcutaneous tissue; M00–M99, musculoskeletal system and connective tissue; and N00–N99, diseases of the genitourinary system. Encounters with primary and secondary diagnoses outside these 14 disease-related chapters were not used to construct trajectories.

Process mining in routine-care data generates many rare trajectories, often with too few observations for stable estimation. Therefore, we prespecified inclusion of trajectories occurring in ≥0.5% of individuals for inferential analyses [11, 22]. Next, we estimated associations with CKD severity for trajectory occurrence and with subsequent all-cause mortality. For each trajectory, occurrence was modelled as a binary indicator (ever/never during follow-up) using modified Poisson regression (log link) with an offset for person-time and adjustment for age, sex, and calendar year; estimates are presented as incidence rate ratios (IRRs). Associations with all-cause mortality are presented as hazard ratios (HRs) from Cox models adjusted for age, sex, and calendar year. We assessed proportional hazards using scaled Schoenfeld residuals and interpreted trajectory-specific HRs as time-averaged relative mortality measures within an exploratory framework. Patients were followed until death, emigration from the region or 31st December 2022. We did not adjust for comorbidities or concomitant medications, as these factors may lie along the disease trajectories that our study aimed to characterize and could therefore obscure the patterns of interest. Accordingly, the analyses are intended to be descriptive rather than causal, and the reported associations should not be interpreted inferentially.

We conducted all the analyses using R (version 4.3.2). The process mining discovery is performed using the bupaverse package [23] in R. The study reported follows the STROBE guideline [24].

## RESULTS

### Patient characteristics

A total of 113 647 patients were hospitalized for a MI or HF in Stockholm during 2006–2021. After applying inclusion and exclusion criteria, 29 901 patients remained in our study. The main reasons for exclusion were death during hospital admission, lack of measurements of creatinine and particularly albuminuria. Of the included patients, 38.5% (11 504/29 901) entered in our study because of an incident MI; the remaining 61.5% (18 397/29 901) because of an incident HF. At baseline, 10 813 (36%) had no-CKD, 4186 (14%) had mild CKD (CKD1–2), and 14 902 (50%) had moderate to severe CKD (CKD3–5, Supplemental Fig. S1).

Patients with more severe CKD were progressively older (median 71, 73, and 82 years for no-CKD, stages 1–2 and 3–5, respectively), had a higher number of comorbid conditions and more often used medications to treat them. As a sole exception, the proportion of statin use was slightly lower in CKD3–5 (44.2%) than in no-CKD (53.0%) (Table 1). Additional comorbid conditions are shown in Supplemental Table S3.

**Table 1:** Baseline characteristics of survivors of an incident MI or HF, stratified by the presence and severity of CKD.

	No CKD (*n* = 10 813)	CKD1–2 (*n* = 4186)	CKD3–5 (*n* = 14 902)
Qualifying entry condition, *n* (%)			
Myocardial infarction	5577 (51.6)	1625 (38.8)	4302 (28.9)
Heart failure	5236 (48.4)	2561 (61.2)	10 600 (71.1)
Age, years			
Median (Q1, Q3)	71 (63, 78)	73 (64, 80)	82 (76, 87)
Mean (SD)	70 (11.2)	71 (11.5)	81 (9.2)
Min, max	18, 102	18, 97	18, 108
Age category, *n* (%)			
18–59 years	1848 (17.1)	621 (14.8)	408 (2.7)
60–79 years	6689 (61.9)	2502 (59.8)	5441 (36.5)
≥80 years	2276 (21.0)	1063 (25.4)	9053 (60.8)
Female, No. (%)	4667 (43.2)	1535 (36.7)	7471 (50.1)
eGFR ml/min/1.73m^2^	73 (66, 82)	72 (65, 81)	45 (32, 53)
Albuminuria category			
A1	10 803 (100.0)	0 (0.0)	8147 (54.7)
A2	0 (0.0)	2954 (70.6)	3805 (25.5)
A3	0 (0.0)	1232 (29.4)	2950 (19.8)
Comorbid conditions, *n* (%)			
Diabetes	4509 (41.7)	2326 (55.6)	6557 (44.0)
Hypertension	8191 (75.8)	3366 (80.4)	13 049 (87.6)
Chronic pulmonary disease	2014 (18.6)	911 (21.8)	2855 (19.2)
Cancer	1131 (10.5)	518 (12.4)	1973 (13.2)
Stroke	1488 (13.8)	770 (18.4)	3272 (22.0)
Depression	1040 (9.6)	391 (9.3)	1211 (8.1)
Ongoing medications, *n* (%)			
ACEI/ARBs	6950 (64.3)	2817 (67.3)	9885 (66.3)
Beta blocking agents	6864 (63.5)	2664 (63.6)	9978 (67.0)
Calcium channel blockers	3104 (28.7)	1546 (36.9)	5857 (39.3)
Diuretics	3875 (35.8)	1880 (44.9)	9441 (63.4)
Statins	5730 (53.0)	2106 (50.3)	6592 (44.2)

Patients were followed for a median of 2.8 (IQR: 1–6) years, during which 19 143 (64%) patients experienced MI/HF readmission and 15 206 (51%) died.

### Indicators of care complexity

Table 2 shows a clear gradient of increasing care complexity and resource utilization with more severe CKD stages. At hospital discharge, patients with more severe CKD had a higher burden of comorbid conditions; e.g. 36% of participants with no-CKD had 5–9 comorbid conditions, compared with 66% and 69% of participants in the groups with CKD1–2 or CKD3–5, respectively. Similarly, polypharmacy was common, and up to 64% of participants with CKD3–5 were dispensing ≥10 distinct medications, compared to 53% in participants with no CKD.

**Table 2:** Indicators of care complexity across the presence and severity of CKD.

Indicator	No-CKD (*n* = 10 813)	CKD1–2 (*n* = 4186)	CKD3–5 (*n* = 14 902)
**A. Baseline indicators of complexity**			
**Number of comorbid conditions at index, %**			
<5	63.2	31.5	29.9
5–9	36.4	66.4	68.5
≥10	0.4	2.1	1.5
**Distinct medications dispensed in the 6 months prior to index, %**			
<5	11.1	10.0	6.0
5–9	36.0	30.8	30.0
≥10	52.9	59.1	64.0
**B. Care Complexity in the First Year Post–MI/HF**			
**Distinct ICD–10 diagnoses issues within a year, % (95% CI)**			
<5	35.5	33.9	32.2
5–9	48.6	46.6	46.6
≥10	15.9	19.5	21.2
**Distinct medications dispensed within a year, % (95% CI)**			
<5	6.8	8.4	9.0
5–9	22.4	17.5	16.6
≥10	70.8	74.1	74.4
**C. Healthcare Utilization metrics during follow-up**			
**Rate of hospital admissions and total number of days spent in hospital per year**			
Incidence rate per 100 person–years (95% CI)	106.9 (106.0–107.8)	146.7 (144.9–148.5)	182.7 (181.5–183.9)
No. of days spent in hospital per year, Median (Q1–Q3)	3.2 (0.3–12.9)	6.5 (0.9–21.9)	10.9 (2.4–31.5)
**Rates of outpatient visits to specialized care**			
Incidence rate per 100 person–years (95% CI)	1260.4 (1257.3–1263.5)	1491.1 (1485.2–1497.0)	1844.6 (1840.7–1848.5)
**Rates of visits to primary care**			
Incidence rate per 100 person–years (95% CI)	3026.5 (3021.7–3031.3)	4345.2 (4313.5–4376.9)	5379.5 (5372.9–5386.1)
**D. Clinical Outcomes**			
**Rates of HF/MI readmission**			
Incidence rate per 100 person–years (95% CI)	128.7 (127.8–129.7)	192.4 (190.3–194.6)	210.0 (208.7–211.3)
**Rates of all-cause death**			
Rate per 100 person–years (95% CI)	7.3 (7.1–7.5)	12.3 (11.8–12.8)	20.1 (19.7–20.5)

During the first year post-MI/HF (Table 2), care complexity in terms of received diagnoses or number of distinct medications dispensed intensified across all groups, but remained highest in patients with CKD: e.g. 21% of patients with CKD3–5 received ≥10 distinct clinical diagnoses versus 16% in the group with no CKD. Supplemental Table S4 reports complementary information on clinical diagnoses and medication dispensations throughout follow-up, describing in general increased rates of diagnoses or drug dispensations across all disease families and therapeutic areas.

Across more severe CKD stages, the use of healthcare resources increased at all levels of care. The rates of hospitalizations increased from 107 per 100 person-years in persons with no-CKD to 183 in persons with CKD3–5, and the median number of days spent in hospital per year of follow-up increased from 3 to 11 days, for no-CKD and CKD3–5, respectively. The rate of visits to outpatient specialist care increased by 46% and the rate of visits to primary care increased by 78% in CKD3–5 compared to no CKD (Table 2).

Across more severe CKD stages, participants experienced worse clinical outcomes, with higher rates of HF/MI readmission (210 events per 100 person-years in CKD3–5, compared to 129 events in the no-CKD group, representing a 63% increase) and all-cause mortality (20 deaths per 100 person-years in CKD3–5 versus 7 in no-CKD, nearly three-fold higher) (Table 2).

### Post-MI/HF disease trajectories

We constructed networks of disease transitions following an incident MI/HF event stratified by the presence and severity of CKD. For hospitalization diagnoses, we identified 8927 unique disease trajectories, and for outpatient diagnoses, we identified 20 805 unique trajectories.

**Figure 1:**
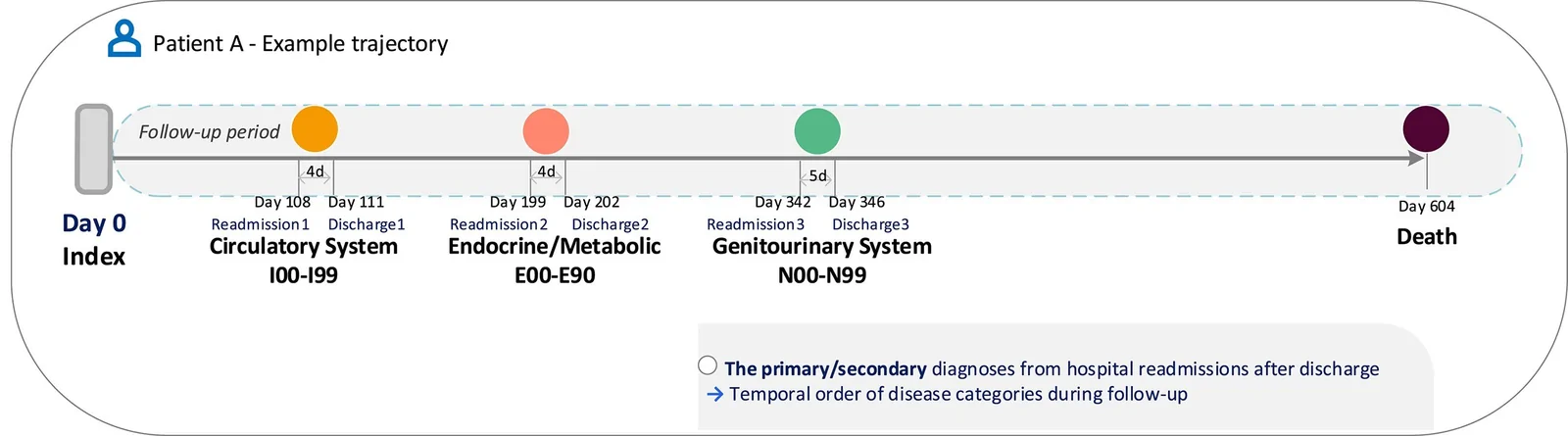
Visual example of a patient trajectory of disease accrual. Day 0 marks the date of discharge from the patient´s MI/HF(index date). A subsequent hospital admission occurred on day 108, attributed to a circulatory system diagnosis (ICD-10 I00–I99; 4-day stay). On day 199, a second hospital admission carried an endocrine/metabolic disease diagnosis (E00–E90; 4-day stay). On day 342, a third hospital admission was attributed to a genitourinary diagnosis (N00–N99). Death occurred 262 days after the last hospital admission.

**Figure 2:**
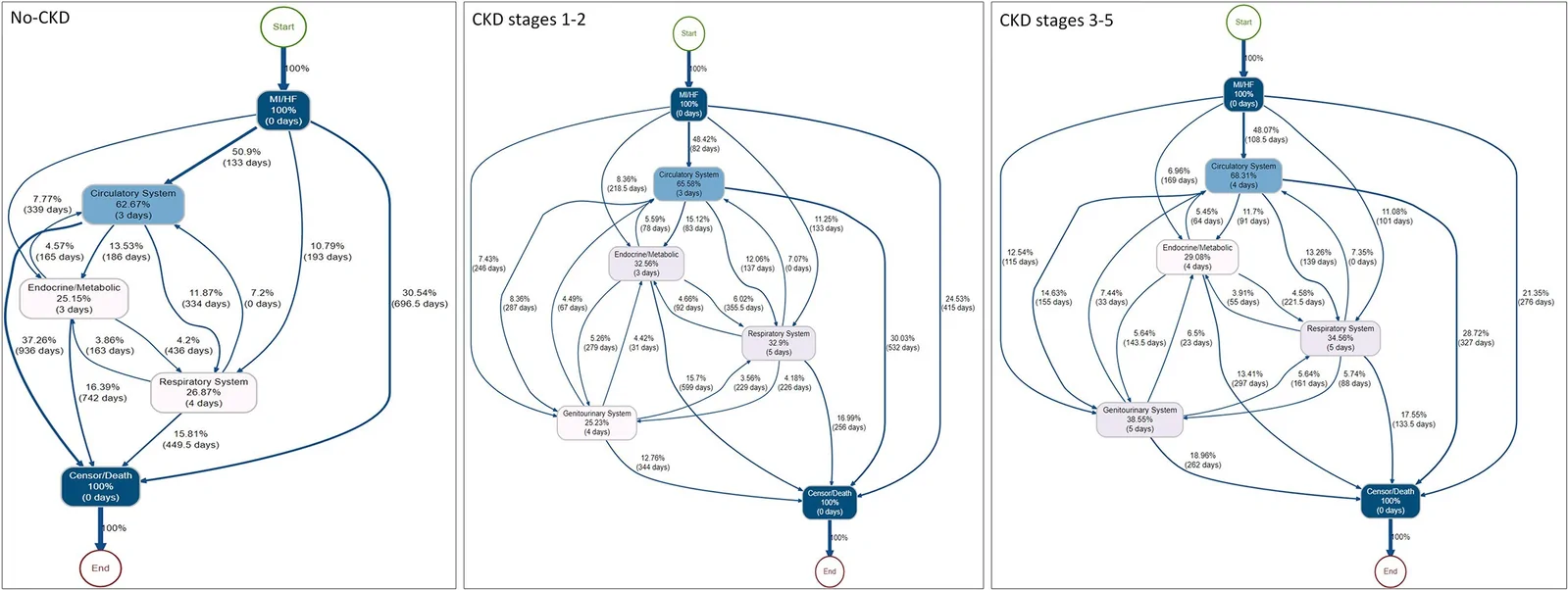
DFG of temporally ordered hospitalization diagnoses after incident MI/HF (Stockholm, 2006–2021), showing the most frequent pathways that together cover 70% of all transitions across participants with no-CKD, CKD stages 1–2, and CKD stages 3–5, separately. Arrows represent immediate A→B transitions; line thickness is proportional to transition frequency. Node labels show relative case frequency and median length of stay (days). Edge labels show relative case (proportion of patients with A directly followed by B) and median inter-event time (days). Nodes are ICD-10 chapter–level diagnoses: infectious/parasitic (A00–B99), neoplasms (C00–D48), blood/immune (D50–D89), endocrine/nutritional/metabolic (E00–E90), mental/behavioural (F00–F99), nervous system (G00–G99), eye/adnexa (H00–H59), ear/mastoid process (H60–H95), circulatory system (I00–I99), respiratory system (J00–J99), digestive system (K00–K93), skin/subcutaneous tissue (L00–L99), musculoskeletal system (M00-–M99), and genitourinary system (N00–N99).

Figure 1 depicts an example of an individual patient’s hospital disease trajectory; the patient’s post-index course included three readmissions. After the index MI/HF discharge (day 0), the first readmission occurred on day 108 for a circulatory system diagnosis (ICD-10 I00–I99; 4-day stay). Ninety-one days later (day 199), the patient was readmitted for an endocrine/metabolic diagnosis (E00–E90; 4-day stay), followed 143 days later (day 342) by a genitourinary diagnosis (N00–N99). The patient died 262 days after the final admission. This patient vignette illustrates the temporal ordering of disease categories that underlie the transitions summarized in the DFG (Fig. 2).

Figure 2 graphically depicts the most frequent disease categories and their sequence of accrual that caused hospital admissions during follow-up. For all CKD categories, circulatory diseases were the most frequent causes of hospital admission (present in 68% of the CKD3–5, 66% of the CKD1–2 group, and 63% of the no-CKD group). In persons with no-CKD, this was followed by both endocrine/metabolic diseases and respiratory diseases. In persons with CKD1–2, the frequency of endocrine/metabolic and respiratory diseases increased, connecting thereafter with hospital admissions due to complications of the genitourinary system. In persons with CKD3–5, respiratory diseases, along with endocrine/metabolic diseases, were instead the most common patterns, followed by complications of the genitourinary system (Fig. 2, Supplemental Table S5). These patterns and sequences were generally similar when looking at consultations in outpatient care (Supplemental Table S5).

#### Hospitalisation trajectories and subsequent mortality

**Figure 3:**
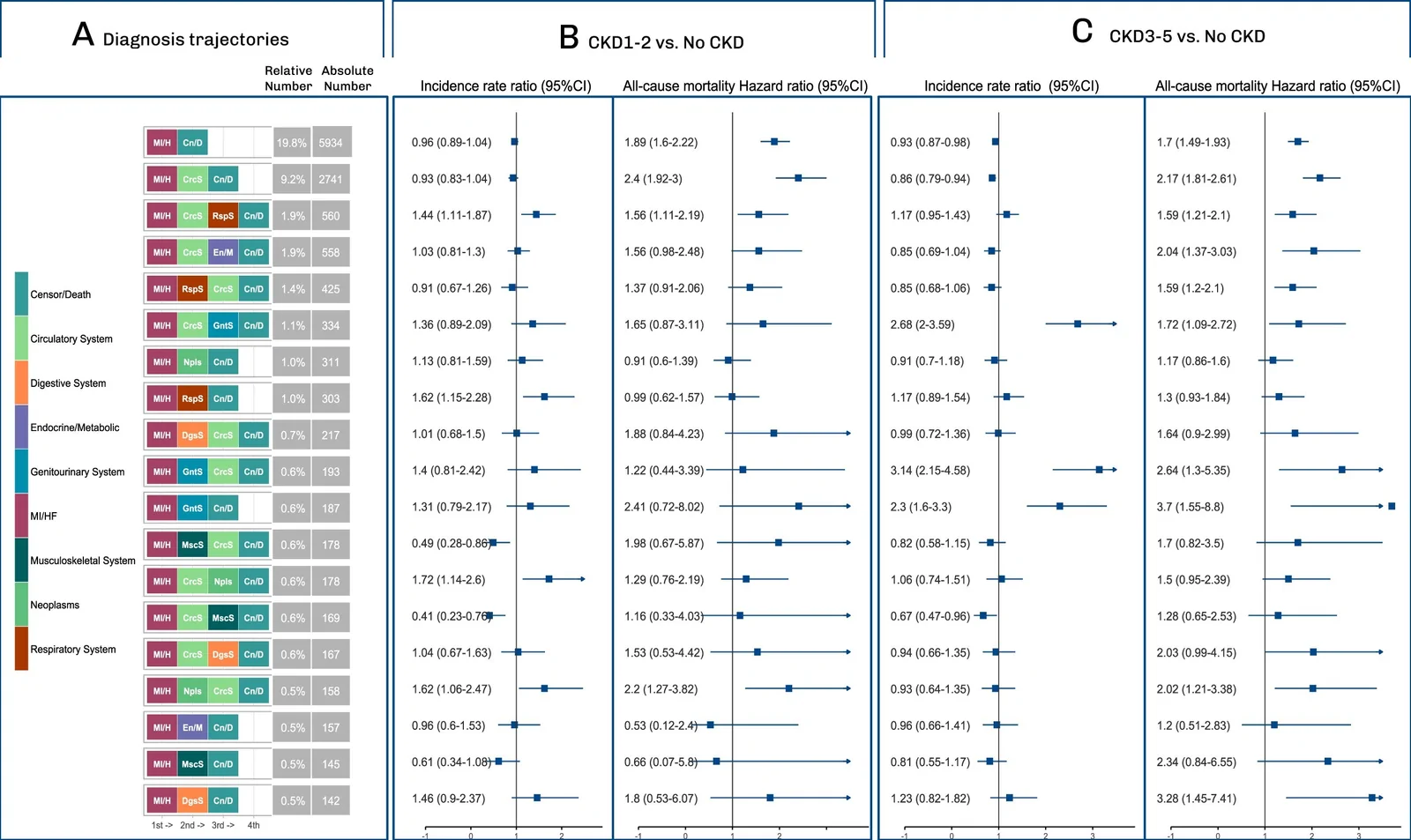
Disease trajectories of primary hospitalization cause for survivors of a MI/HF stratified by the presence and severity of CKD in the region of Stockholm, Sweden, during 2006–2021. Panel a shows the most common disease trajectories (occurring in ≥0.5% of the cohort) by descending frequency. Each row represents one trajectory that patients experienced after the index MI/HF event. Each row of panels b and c corresponds to the trajectory shown in the same row of panel a; readers can therefore scan horizontally across the figure to obtain, for each trajectory, both its CKD-specific frequency and its associated hazard of mortality. IRRs describe the occurrence of each trajectory in CKD1–2 and CKD3–5, respectively, versus no-CKD. An IRR >1 indicates that, given equal observation time, patients with CKD were more likely to experience that specific disease trajectory than patients without CKD. HRs describe the rates of all-cause mortality among patients experiencing each trajectory in CKD1–2 and CKD3–5, respectively, versus no-CKD. Squares represent point estimates and horizontal bars represent 95% confidence intervals. The vertical reference line at 1.0 corresponds to the no-CKD comparator; estimates falling to the right indicate higher mortality rates.

Figure 3 focuses on the most common disease trajectories (occurring in >0.5% of the population) that resulted in a hospital stay, and evaluates both the probability of being experienced by patients with CKD and their associated probability of dying. We observed that disease trajectories involving a hospitalization due to circulatory diseases were the most common. Furthermore, compared to the no-CKD group, both CKD groups had a significantly higher hazard of death after experiencing hospital admission due to circulatory diseases (HR: 2.40, 95% CI 1.92–3.00 for CKD 1–2 and HR: 2.17, 95% CI 1.81–2.61 for CKD3–5) (Fig. 3, Supplemental Fig. S2). Supplemental Table S6 lists the most issued ICD-10 diagnoses across the presence and severity of CKD. For circulatory diseases, diagnoses of HF, hypertension, and atrial fibrillation were the most frequent, and their proportion increases with more severe stages of CKD.

#### High-risk hospitalization trajectories

Figure 3 also identifies some hospitalization trajectories specific to the stages of CKD. Compared to no-CKD, patients with CKD1–2 had a significantly higher probability of progressing from circulatory diseases to respiratory diseases (IRR 1.44, 95% CI 1.11–1.87) and from circulatory diseases to neoplasms (IRR 1.72, 95% CI 1.14–2.60). These specific sequences, [MI/HF→Circulatory→Respiratory] and [MI/HF→Neoplasm→Circulatory], were associated with increased mortality (HR: 1.56, 95% CI 1.11–2.19 and HR: 2.20, 95% CI 1.27–3.82, respectively). The most diagnosed respiratory diseases were pneumonia and chronic obstructive pulmonary disease, which increased in frequency with more severe CKD (Table S6). The most diagnosed neoplasms in people with CKD involved prostate cancer and malignant neoplasms of skin (Table S6).

Compared to no-CKD, patients with CKD 3–5 showed a higher probability of progressing from circulatory diseases to genitourinary diseases, and vice versa [MI/HF→Circulatory→Genitourinary or MI/HF→Genitourinary→Circulatory] (IRR 2.68, 95% CI range: 2.00–3.59). Both these trajectories were associated with higher mortality hazards. The most issued diagnoses of the genitourinary system for people with CKD were, not unexpectedly, a CKD diagnosis but also cystitis and acute kidney injury (AKI, Table S6).

#### High-risk outpatient-specialist trajectories

**Figure 4:**
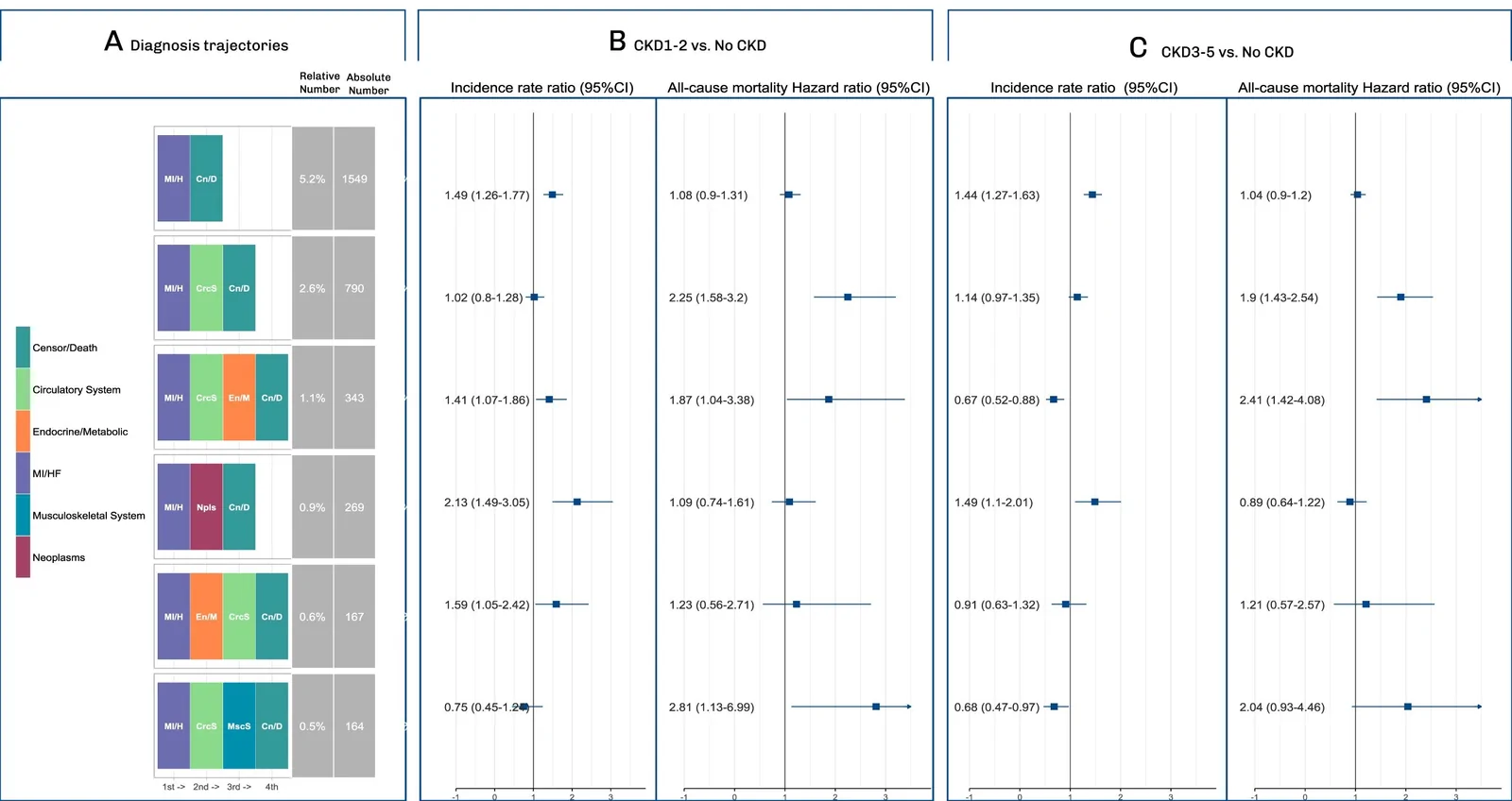
Disease trajectories of primary outpatient-specialist consultations for survivors of a MI/HF stratified by the presence and severity of CKD in the region of Stockholm, Sweden, during 2006–2021. Panel a shows the most common disease trajectories (occurring in ≥0.5% of the cohort) by descending frequency. Each row represents one trajectory that patients experienced after the index MI/HF event. Each row of panels b and c corresponds to the trajectory shown in the same row of panel a; readers can therefore scan horizontally across the figure to obtain, for each trajectory, both its CKD-specific frequency and its associated hazard of mortality. IRRs describe the occurrence of each trajectory in CKD1–2 and CKD3–5, respectively, versus no-CKD. An IRR >1 indicates that, given equal observation time, patients with CKD were more likely to experience that specific disease trajectory than patients without CKD. HRs describe the rates of all-cause mortality among patients experiencing each trajectory in CKD1–2 and CKD3–5, respectively, versus no-CKD. Squares represent point estimates and horizontal bars represent 95% confidence intervals. The vertical reference line at 1.0 corresponds to the no-CKD comparator; estimates falling to the right indicate higher mortality rates.

Figure 4 evaluates through process mining the trajectories of disease diagnosis and accrual through outpatient-specialist consultations, observing risk patterns complementary to the evaluation of hospital admissions (Supplemental Fig. S3).

Compared to no-CKD, patients with CKD1–2 were associated with higher rates of trajectories involving endocrine/metabolic consultations (IRR: 1.41, 95% CI 1.07–1.86 and IRR: 1.59, 95% CI 1.05–2.42), and these trajectories were associated with higher mortality hazards. The most common diagnoses in this trajectory involved consultations regarding type 2 diabetes (Table S6). CKD1–2 was also associated with higher rates of trajectories involving cancer consultations (IRR: 2.13, 95% CI 1.49–3.05).

Compared to no-CKD, patients with CKD3–5 were less likely to experience health trajectories involving endocrine/metabolic disease; however, among those with such trajectories, the hazard of death was higher (HR: 2.41, 95% CI 1.42–4.08). In addition to type 2 diabetes-related consultations, consultations for disorders of fluid, electrolyte, and acid-base balance were more frequent (Table S6). Patients with CKD3–5 were also associated with higher rates of trajectories involving cancer consultations (IRR: 1.49, 95% CI 1.10–2.01).

Figures 3 and 4 showed that 19.8% of the cohort were censored or died without any defined in-hospital events, and 5.2% without any outpatient events. To determine whether these patients survived the median follow-up period or died, we stratified the MI/HF-to-death/censoring trajectory by the study median follow-up time (2.8 years) (Supplemental Table S7).

## DISCUSSION

In this large, routine care, observational study of patients surviving MI or HF, we quantified the complexity and healthcare resource utilization of individuals with comorbid CKD. With advancing CKD stage, patients exhibited greater multimorbidity, more frequent polypharmacy, broader and more frequent use of healthcare services, as well as worse clinical outcomes. Process mining of disease trajectories revealed distinct patterns of disease accrual, with circulatory diseases featuring the most, but becoming progressively more common across more severe CKD. Within these trajectories, and compared to those with no-CKD, patients with CKD1–2 and CKD3–5 more frequently experienced progression to endocrine/metabolic diseases, respiratory diseases, neoplasia, and genitourinary diseases. These trajectories were also associated with increased death hazards. Collectively, this study has implications by illustrating the complex course of illness among patients with both CVD and CKD, while also identifying actionable insights to support clinicians and patients in foreseeing disease evolution and developing tailored care preventive pathways.

We observed substantial differences in the average clinical complexity of patients with MI or HF who also had CKD. The administrative nature of our data allowed us to capture medical dimensions of complexity, which may be interpreted as reflections of the workload associated with routine clinical practice. However, we lacked information on additional factors that contribute to complex care, such as socioeconomic circumstances, health literacy, and care coordination [25]. Recognizing and quantifying the complexity of CVD patients with comorbid CKD has implications. The perception that these patients are ‘too complex to manage’ has given rise to the concept of *renalism*—a historically inappropriate reluctance or refusal to offer indicated diagnostic or therapeutic procedures to patients with CKD due to concerns about potential harm or futility. Yet, prior evidence consistently shows that patients with CKD derive at least comparable, and often greater, absolute benefits from guideline-recommended cardiovascular therapies than those without CKD [26, 27]. In light of the complexity we describe, our findings underscore the importance of improving cardiologists’ awareness and understanding of the bidirectional interplay between cardiac and kidney diseases [28, 29] and support the value of multidisciplinary cardiorenal teams or shared decision-making models involving nephrologists [30, 31]. Finally, in health systems, resource allocation is typically determined by quantifiable elements such as the number of patients, diagnoses, or documented data points—metrics that inadequately capture true clinical complexity [9, 32]. Adjusting resource allocation and planning models to the time and resources required to care for patients at the highest risk of adverse outcomes would be both clinically and economically justified.

Using process mining, we generated a novel, data-driven visualization of patient trajectories after MI or HF [13]. Consistent with previous reports [11, 33], we observed a high rate of rehospitalization for additional circulatory diseases. Our study adds the new observation that these complications become progressively more common with advancing CKD stage and are strongly linked to subsequent mortality. The higher rate of additional cardiovascular conditions in CKD may be associated with both pathophysiological effects, such as accelerated vascular ageing, chronic inflammation, dysregulated mineral metabolism [34] and cardiac dysfunction/remodelling predisposing to atrial fibrillation or HF [35], and shared risk factors, including smoking, hyperlipidaemia, and hypertension, all of which are important causes and determinants of CKD severity [36]. Importantly, the fact that these disease trajectories emerged in our analysis despite the use of modern, guideline-directed therapies, highlights the potential need for more intensive risk factor management and novel secondary prevention strategies in people with CKD to mitigate the burden of recurrent cardiovascular events and premature death following MI or HF.

Beyond the excess accrual of circulatory diseases, our trajectories revealed that endocrine/metabolic disorders, respiratory diseases, and cancers became increasingly common with advancing CKD. These patterns have strong biological plausibility. Diabetes mellitus was a predominant cause of outpatient specialist consultations among patients with CKD in our study. Diabetes is indeed a major driver of the CKD epidemic, and CKD, in turn, amplifies the risk of diabetes-related complications [37]. Consultations related to disorders in fluid, electrolyte and acid-base balance were common in advanced CKD, as these are a direct consequence of impaired kidney elimination [38], which increases the risk of medication-related adverse events, particularly hyperkalemia, in secondary CVD care [39, 40]. CKD is associated with the risk of cancer, with stronger associations for albuminuria than for eGFR [41–43]. The more frequent diagnoses of prostate cancer and malignant melanoma with advancing CKD observed in our study align with previous evidence [41–43] and may highlight opportunities for targeted screening and early detection. Impaired immune responses due to the accumulation of uremic toxins [44, 45], likely underlie the well-established higher incidence of infections in people with CKD [46–48], particularly respiratory tract infections such as pneumonia, supporting the implementation of tailored vaccination strategies in this population. The emergence of COPD diagnoses in more advanced CKD stages in our study is noteworthy. This association may reflect shared pathophysiological mechanisms (e.g. systemic inflammation), overlapping risk factors (e.g. advanced age, diabetes, hypertension, and smoking), or direct renal–pulmonary interactions mediated by altered vascular tone, fluid balance, and ventilatory control [49, 50].

As expected, consultations to nephrology and diagnoses of CKD became progressively more common with worsening kidney function, consistent with the under-recognition and under-detection of this largely asymptomatic disease and the prevailing care model in which CKD is managed in primary care until advanced stages prompt referral. An interesting finding was the higher accrual of AKI episodes among these patients, which again underscores opportunities for prevention in secondary CVD care. ‘Sick day rules’ [51, 52], available in some healthcare systems, provide, for instance, a practical tool for patients to identify and temporarily discontinue AKI-exacerbating medications (e.g. ACE inhibitors, ARBs, diuretics, renin inhibitors, or NSAIDs, all frequent in secondary CVD care) during periods of acute illness.

Collectively, we believe that the frequency and mortality risks associated with these disease trajectories offer valuable insights for clinicians and policymakers. They underscore the need for anticipatory, integrated care strategies to address the evolving health needs of patients with CKD who experience MI or HF, and to prioritize preventive interventions along the most lethal pathways. The trajectory groups and healthcare-utilization patterns identified here may also provide a foundation for future health-economic evaluations.

A major strength of this study lies in its complete and comprehensive health-data coverage for an entire region and long-term follow-up. Another important asset is the objective ascertainment of CKD stages using laboratory measurements of eGFR and albuminuria. The application of process mining enhanced our analysis by incorporating temporal structure, allowing us to move beyond static prevalence estimates and uncover CKD-specific diagnostic pathways and their timing.

Several limitations should, however, be acknowledged. First, as an observational study, causal inference cannot be drawn, and our descriptive trajectories are intended to support hypothesis generation and inform pathway-oriented care. Second, patients without available creatinine/albuminuria measurements at the time of MI or HF admission were excluded by design; because testing for kidney function measures, particularly albuminuria [53], is performed selectively in clinical practice, our results may not fully generalize to the broader MI/HF population [54, 55]. In this sense, our dataset represents a defined population (citizens of the region of Stockholm) and covers a defined period (2006–2021) with calls for caution when extrapolating to other regions, periods or ethnic diversities. Third, CKD status was defined from a single pre-index measurement of eGFR and albuminuria, which may infer misclassification bias [56]. Fourth, participants entered the cohort at first MI/HF discharge and had different follow-up durations; while time-to-event models account for censoring, longer observation still offers more opportunity for complex trajectory accrual. Fifth, trajectory-specific analyses involve multiple correlated comparisons; because trajectories share overlapping subsequences, these results should be interpreted as exploratory. Sixth, as in all observational studies, we are impacted by residual confounding: diagnoses in administrative data are susceptible to coding misclassification and affected by reimbursement processes; we lacked information on socioeconomic and lifestyle factors, and also on decisions regarding palliative or conservative care. The latter may have influenced our findings in three ways: healthcare utilization in CKD3–5 may partly reflect end-of-life care (e.g. more inpatient days and fewer outpatient specialist visits) rather than active management of newly accruing comorbidities; trajectory patterns may appear truncated or simplified if care shifted towards symptom-focused management; and higher mortality associated with some CKD3–5 trajectories may partly reflect end-of-life transitions rather than newly emerging complications. Finally, process mining prioritizes pathways that patients have in common, so rarer but clinically relevant trajectories may be under-represented. All in all, our trajectories should be interpreted as descriptive of the totality of care pathways observed, which will inevitably blend active management with conservative and palliative care.

To conclude, using a systemiatic, data-driven, and hypothesis-free methodology, this study quantifies real-world care complexity and post-MI/HF disease trajectories of patients with CKD. By moving beyond cross-sectional multimorbidity analyses and quantifying the relative risks of disease pathways, we have identified possible targets for secondary prevention. Our study suggests that a focus on reducing the endocrine, respiratory and cancer disease burden of patients with CKD following MI/HF, may minimize increased dependency and premature mortality. Further understanding of the factors contributing to these trajectories is required to facilitate identification of patients who would benefit the most from closer follow-up and prompt intervention.

## Supplementary Material

sfag242_Supplemental_File

## Data Availability

The data underlying this article cannot be shared publicly due to the privacy of individuals who participated in the study. The data may be shared on reasonable request for academic research collaborations that fulfil GDPR as well as national and institutional ethics regulations and standards by contacting Prof. Juan-Jesus Carrero (juan.jesus.carrero@ki.se).
