# Supplementary material for: Mapping the complexity of care in patients with heart disease living with chronic kidney disease
Source: Clin Kidney J. 2026 Aug 7;19(9):sfag242. doi: 10.1093/ckj/sfag242 (PMC13529821; doi:10.1093/ckj/sfag242)
Supplement: Supplementary file 1 [file sfag242_supplemental_file.docx]

**Supplementary Materials**

Contents

[Figure S1. Flowchart of study cohort selection. Inclusion and exclusion of patients with a first hospitalisation for myocardial infarction or heart failure (2006–2021)	2](#_Toc220961368)

[Table S1. KDIGO Classification of Chronic Kidney Disease (CKD) based on eGFR and Albuminuria Categories^1^	3](#_Toc220961369)

[Table S2. Definitions of Chronic comorbid conditions and ongoing medications	4](#_Toc220961370)

[Table S3. Comorbid conditions at Index stratified by chronic kidney-disease stage, prevalence (n, %)	6](#_Toc220961371)

[Table S4. Mortality, follow-up duration, and incidence of diagnoses issued in connection to hospital-admissions or medications dispensed, stratified by the presence and severity of CKD.	7](#_Toc220961372)

[Table S5. Most frequent diagnoses (at ICD-10 chapter level) issued in outpatient-specialist consultations and during Hospitalisation, stratified by the presence and severity of CKD.	8](#_Toc220961373)

[Table S6*.* The Top Five Most common ICD-10 Diagnoses (with 2-digit level) issued during hospital admissions per each ICD-10 chapter, Stratified by the presence and severity of CKD	9](#_Toc220961374)

[Figure S2 Disease trajectories of primary hospitalization cause for survivors of a MI/HF stratified by the presence and severity of CKD in the region of Stockholm, Sweden, during 2006-2021. Disease trajectory panel (on the left) shows the most frequent chronological disease trajectories related to hospital admissions and ascertained by primary diagnoses codes according to ICD-10 chapter headings. Trajectories are ordered by frequency and only list those occurring in ≥0.5% of cases. The right column summarises the frequency and relative frequency in each trajectory across CKD groups.	13](#_Toc220961375)

[Figure S3 Disease trajectories of primary outpatent-specialist consultation cause for survivors of a MI/HF stratified by the presence and severity of CKD in the region of Stockholm, Sweden, during 2006-2021. Disease trajectory panel (on the left) shows the most frequent chronological disease trajectories related to hospital admissions and ascertained by primary diagnoses codes according to ICD-10 chapter headings. Trajectories are ordered by frequency and only list those occurring in ≥0.5% of cases. The right column summarises the frequency and relative frequency in each trajectory across CKD groups.	14](#_Toc220961376)

[STROBE Statement—Checklist of items that should be included in reports of cohort studies	15](#_Toc220961377)

## Figure S1. Flowchart of study cohort selection. Inclusion and exclusion of patients with a first hospitalisation for myocardial infarction or heart failure (2006–2021)

## Table S1. KDIGO Classification of Chronic Kidney Disease (CKD) based on eGFR and Albuminuria Categories^1^

| KDIGO **CKD Stage** | **eGFR Category (ml/min/1.73 m^2^)** | **Albuminuria Category (A)** | **CKD group in this study** |
| --- | --- | --- | --- |
| No CKD | G1 (≥90) or G2 (60-89) | A1(<30mg/g or <3mg/mmol) | No CKD (CKD0) |
| CKD Stage 1 | G1 | A2 (30-300mg/g or 3-30mg/mmol) or A3 (>300mg/g or >30mg/mmol)) | Mild CKD (CKD1-2) |
| CKD Stage 2 | G2 | A2 or A3 | Mild CKD (CKD1-2) |
| CKD Stage 3 | G3a (45-59) or G3b (30-44) | Any (A1–A3) | Moderate to Severe CKD (CKD3-5) |
| CKD Stage 4 | G4 (15-29) | Any (A1–A3) | Moderate to Severe CKD (CKD3-5) |
| CKD Stage 5 | G5 (<15) | Any (A1–A3) | Moderate to Severe CKD (CKD3-5) |

^1^KDIGO 2024 Clinical Practice Guideline for the Evaluation and Management of Chronic Kidney Disease

## Table S2. Definitions of Chronic comorbid conditions and ongoing medications

| **Comorbid conditions** | **ICD-10 codes** |
| --- | --- |
| Myocardial infarction | I21, I22 |
| Heart failure | I099, I110, I130, I132, I255, I420, I425, I426, I427, I428, I429, I43, I50 |
| Hypertension | I10-I13, I15 |
| Diabetes | E10-E14 |
| Chronic Pain | F45.4, G89.0, G89.2, G89.4, M08.1, M25.50, M25.51, M25.55, M25.56, M25.57, M43.2, M43.3, M43.4, M43.5, M43.6, M45, M46.1, M46.3, M46.4, M46.9, M47, M48.0, M48.1, M48.8, M48.9, M50.8, M50.9, M51, M53.1, M53.2, M53.3, M53.8, M53.9, M54, M60.8, M60.9, M63.3, M79.0, M79.1, M79.2, M79.6, M79.7, M96.1 |
| Stroke | G45.0-G45.3, G45.8-G45.9, H34.1, I60, I61, I63, I64 |
| Chronic Pulmonary Disease | I27.8, I27.9, J40–J44, J46-J47, J60–J67, J68.4, J70.1, J70.3 |
| Hypothyroidism | E00–E03, E89.0 |
| Rheumatoid arthritis | M05, M06, M31.5, M32–M34, M35.1, M35.3, M36.0 |
| Cancer | C81–C85, C88, C90.0, C90.2, C96, C77–C80, C18-C21, C33-C34, C38.4, C45.0, C46.71, C50, C53, C61, D01.0-D01.3, D02.2, D05-D06, D07.5 |
| Asthma | J45 |
| Atrial fibrillation | I48.0 |
| Constipation | K55.8, K56.0, K56.4, K56.7, K59.0, K63.1, K63.4, K63.81, K63.88, K92.80, K92.88 |
| Peripheral vascular disease | I70.2 |
| Depression | F20.4, F31.3–F31.5, F32, F33, F34.1, F41.2, F43.2 |
| Dementia | F00–F03, F05.1, G30, G31.1 |
| Psoriasis | L40.0, L40.1, L40.2, L40.3, L40.4, L40.8, L40.9 |
| Irritable bowel syndrome | K58 |
| Cirrhosis | K70.3, K74.3, K74.4, K74.5, K74.6, I85.0, I85.9, I98.2, I98.3, K65.0, K65.8, K65.9, K67.0, K67.1, K67.2, K67.3, K67.8, K76.7, K93.0, R18 |
| Peptic ulcer disease | K25.7, K25.9, K26.7, K26.9, K27.7, K27.9, K28.7, K28.9 |
| Epilepsy | G40-G41 |
| Inflammatory bowel disease | K50, K51 |
| Alcohol misuse | E52, F10, G62.1, I42.6, K29.2, K70.0, K70.3, K70.9, T51, Z50.2, Z71.4, Z72.1 |
| Parkinson | G20, G21, G22 |
| Schizophrenia | F20, F21, F23.2, F25 |
| Multiple sclerosis | G35, G36, G37, H46 |
| Chronic viral hepatitis B | B16, B18.0-B18.1 |
| **Ongoing Medications** | **ATC codes** |
| ACEI/ARBs | C09 |
| Beta blocking agents | C07 |
| Calcium channel blockers | C08 |
| Diuretics | C03 |
| Statins | C10AA |

## Table S3. Comorbid conditions at Index stratified by chronic kidney-disease stage, prevalence (n, %)

|  | No CKD | CKD1-2 | CKD3-5 |
| --- | --- | --- | --- |
| CKD | 0 (0.0%) | 4186 (100.0%) | 14902 (100.0%) |
| Hypertension | 8191 (75.8%) | 3366 (80.4%) | **13049 (87.6%)** |
| Heart Failure | 5887 (54.4%) | 2823 (67.4%) | **11573 (77.7%)** |
| Diabetes | 4509 (41.7%) | **2326 (55.6%)** | 6557 (44.0%) |
| Chronic Pain | **4765 (44.1%)** | 1658 (39.6%) | 6474 (43.4%) |
| Myocardio Infarction | **6052 (56.0%)** | 1846 (44.1%) | 5057 (33.9%) |
| Stroke | 1488 (13.8%) | 770 (18.4%) | **3272 (22.0%)** |
| Chronic Pulmonary Disease | 2014 (18.6%) | **911 (21.8%)** | 2855 (19.2%) |
| Hypothyroidism | 1228 (11.4%) | 437 (10.4%) | **2111 (14.2%)** |
| Rheumatoid  arthritis | 1185 (11.0%) | 348 (8.3%) | **2011 (13.5%)** |
| Cancer | 1131 (10.5%) | 518 (12.4%) | **1973 (13.2%)** |
| Asthma | **1633 (15.1%)** | 529 (12.6%) | 1918 (12.9%) |
| Atrial fibrillation | 806 (7.5%) | 355 (8.5%) | **1530 (10.3%)** |
| Constipation | 757 (7.0%) | 328 (7.8%) | **1379 (9.3%)** |
| Peripheral vascular disease | 475 (4.4%) | 328 (7.8%) | **1338 (9.0%)** |
| Depression | **1040 (9.6%)** | 391 (9.3%) | 1211 (8.1%) |
| Dementia | 350 (3.2%) | 193 (4.6%) | **1046 (7.0%)** |
| Psoriasis | 648 (6.0%) | **284 (6.8%)** | 747 (5.0%) |
| Irritable bowel syndrome | **522 (4.8%)** | 154 (3.7%) | 585 (3.9%) |
| Cirrhosis | 276 (2.6%) | **176 (4.2%)** | 480 (3.2%) |
| Peptic ulcer disease | 244 (2.3%) | **110 (2.6%)** | 365 (2.4%) |
| Epilepsy | 256 (2.4%) | **141 (3.4%)** | 318 (2.1%) |
| Inflammatory bowl disease | **204 (1.9%)** | **79 (1.9%)** | 273 (1.8%) |
| Alcohol misuse | 463 (4.3%) | **234 (5.6%)** | 269 (1.8%) |
| Parkinson | 139 (1.3%) | **64 (1.5%)** | 193 (1.3%) |
| Schizophrenia | 93 (0.9%) | **43 (1.0%)** | 55 (0.4%) |
| Multiple sclerosis | **51 (0.5%)** | **22 (0.5%)** | 41 (0.3%) |
| Chronic viral hepatitis B | 59 (0.5%) | **33 (0.8%)** | 41 (0.3%) |

## Table S4. Mortality, follow-up duration, and incidence of diagnoses issued in connection to hospital-admissions or medications dispensed, stratified by the presence and severity of CKD.

|  | CKD stage 0 (n=10,813) | CKD stage 1-2 (n=4,186) | CKD stage 3-5 (n=14,902) |
| --- | --- | --- | --- |
| **All-cause mortality, cases (per 100 person-years)** | 3775 (7.3) | 2031 (12.3) | 9400 (20.1) |
| **Follow-up duration (from Index date for cohorts) (years), median [Q1-Q3]** | 3.8 (1.4, 7.2) | 2.9 (1.0, 5.9) | 2.3 (0.7, 4.7) |
| **Incident diagnoses (ICD-10 chapter) post MI/HF, n (rate per 100 person-years):** |  |  |  |
| Infectious/Parasitic (A00-B99) | 3892 (7.6) | 1495 (9) | 5130 (11) |
| Neoplasms (C00-D48) | 3687 (7.2) | 1451 (8.8) | 5172 (11.1) |
| Blood/Immune (D50-D89) | 2369 (4.6) | 1022 (6.2) | 3922 (8.4) |
| Endocrine/Nutritional/Metabolic (E00-E90) | 6650 (13) | 2743 (16.6) | 8220 (17.6) |
| Mental/Behavioural (F00-F99) | 3578 (7) | 1317 (8) | 4276 (9.1) |
| Nervous System (G00-G99) | 3422 (6.7) | 1217 (7.3) | 3666 (7.8) |
| Eye/Adnexa (H00-H59) | 4630 (9) | 1667 (10.1) | 5605 (12) |
| Ear/Mastoid Process (H60-H95) | 3461 (6.8) | 1140 (6.9) | 4251 (9.1) |
| Circulatory System (I00-I99) | 9956 (19.5) | 3793 (22.9) | 13351 (28.6) |
| Respiratory System (J00-J99) | 5733 (11.2) | 2248 (13.6) | 7500 (16) |
| Digestive System (K00-K93) | 4573 (8.9) | 1625 (9.8) | 5737 (12.3) |
| Skin/Subcutaneous Tissue (L00-L99) | 4585 (9) | 1662 (10) | 5810 (12.4) |
| Musculoskeletal System (M00-M99) | 6920 (13.5) | 2360 (14.2) | 8149 (17.4) |
| Genitourinary System (N00-N99) | 4941 (9.7) | 2074 (12.5) | 9370 (20) |
| **Incident medication users post MI/HF, n (incidence rate per 100 person-years):** |  |  |  |
| A - Alimentary tract and metabolism | 9523 (18.6) | 3706 (22.4) | 13005 (27.8) |
| B - Blood and blood forming organs | 9826 (19.2) | 3690 (22.3) | 13140 (28.1) |
| C - Cardiovascular system | 10220 (20) | 3880 (23.4) | 13764 (29.4) |
| D - Dermatologicals | 6031 (11.8) | 2317 (14) | 8383 (17.9) |
| G - Genitourinary system and sex hormones | 4305 (8.4) | 1514 (9.1) | 4738 (10.1) |
| H - Systemic hormonal preparations | 4174 (8.2) | 1489 (9) | 5988 (12.8) |
| J - Antiinfectives for systemic use | 7245 (14.2) | 2798 (16.9) | 9322 (19.9) |
| L - Antineoplastic and immunomodulating agents | 1576 (3.1) | 548 (3.3) | 2041 (4.4) |
| M - Musculoskeletal system | 4885 (9.5) | 1682 (10.2) | 5915 (12.7) |
| N - Nervous system | 8881 (17.4) | 3468 (20.9) | 12324 (26.4) |
| P - Antiparasitic products, insecticides and repellents | 1030 (2) | 380 (2.3) | 1177 (2.5) |
| R - Respiratory system | 6659 (13) | 2481 (15) | 8162 (17.5) |
| S - Sensory organs | 4851 (9.5) | 1693 (10.2) | 6143 (13.1) |
| V - Various | 151 (0.3) | 138 (0.8) | 1171 (2.5) |

Clinical diagnoses follow the ICD-10^th^ revision Chapter headings, and medications follow the classification of the ATC chapters

## Table S5. Most frequent diagnoses (at ICD-10 chapter level) issued in outpatient-specialist consultations and during Hospitalisation, stratified by the presence and severity of CKD.

| **Event** | **No CKD, n (%)** | | **CKD1-2, n (%)** | | **CKD3-5, n (%)** | |
| --- | --- | --- | --- | --- | --- | --- |
| **Outpatient-specialist consultations** | | | | | | |
| Circulatory System (I00-I99) | 9743 | 90.1 | 3654 | 87.3 | 12523 | 84.0 |
| Musculoskeletal System (M00-M99) | 6810 | 63.0 | 2299 | 54.9 | 7813 | 52.4 |
| Endocrine/Metabolic (E00-E90) | 6683 | 61.8 | 2703 | 64.6 | 7959 | 53.4 |
| Respiratory System (J00-J99) | 5016 | 46.4 | 1836 | 43.9 | 5526 | 37.1 |
| Eye/Adnexa (H00-H59) | 4626 | 42.8 | 1664 | 39.8 | 5593 | 37.5 |
| Skin/subcutaneous Tissue (L00-L99) | 4505 | 41.7 | 1611 | 38.5 | 5637 | 37.8 |
| Genitourinary System (N00-N99) | 4487 | 41.5 | 1839 | 43.9 | 8315 | 55.8 |
| Digestive System (K00-K93) | 4160 | 38.5 | 1386 | 33.1 | 4722 | 31.7 |
| Neoplasms (C00-D48) | 3542 | 32.8 | 1379 | 32.9 | 4842 | 32.5 |
| Ear/Mastoid (H60-H95) | 3431 | 31.7 | 1124 | 26.9 | 4204 | 28.2 |
| Mental/Behavioural (F00-F99) | 3332 | 30.8 | 1204 | 28.8 | 3720 | 25.0 |
| Nervous System (G00-G99) | 3196 | 29.6 | 1100 | 26.3 | 3246 | 21.8 |
| Infectious/Parasitic (A00-B99) | 3102 | 28.7 | 1069 | 25.5 | 3430 | 23.0 |
| Blood/Immune (D50-D89) | 2004 | 18.5 | 856 | 20.4 | 3104 | 20.8 |
| **Hospitalisations** | | | | | | |
| Circulatory System (I00-I99) | 6776 | 62.7 | 2745 | 65.6 | 10180 | 68.30 |
| Respiratory System (J00-J99) | 2905 | 26.9 | 1377 | 32.9 | 5150 | 34.60 |
| Endocrine/Metabolic (E00-E90) | 2720 | 25.2 | 1363 | 32.6 | 4333 | 29.10 |
| Digestive System (K00-K93) | 2093 | 19.4 | 862 | 20.6 | 3286 | 22.10 |
| Genitourinary System (N00-N99) | 2005 | 18.5 | 1056 | 25.2 | 5744 | 38.50 |
| Musculoskeletal System (M00-M99) | 1976 | 18.3 | 692 | 16.5 | 2854 | 19.2 |
| Infectious/Parasitic (A00-B99) | 1767 | 16.3 | 853 | 20.4 | 3264 | 21.9 |
| Neoplasms (C00-D48) | 1596 | 14.8 | 716 | 17.1 | 2395 | 16.1 |
| Mental/Behavioural (F00-F99) | 1259 | 11.6 | 526 | 12.6 | 2011 | 13.5 |
| Nervous System (G00-G99) | 1110 | 10.3 | 445 | 10.6 | 1302 | 8.7 |
| Blood/Immune (D50-D89) | 982 | 9.1 | 453 | 10.8 | 1965 | 13.2 |
| Skin/subcutaneous Tissue (L00-L99) | 581 | 5.4 | 333 | 8.0 | 1104 | 7.4 |
| Ear/Mastoid (H60-H95) | 142 | 1.3 | 40 | 1.0 | 163 | 1.1 |
| Eye/Adnexa (H00-H59) | 137 | 1.3 | 73 | 1.7 | 200 | 1.3 |

## Table S6*.* The Top Five Most common ICD-10 Diagnoses (with 2-digit level) issued during hospital admissions per each ICD-10 chapter, Stratified by the presence and severity of CKD

| No CKD | | |  | CKD1-2 | | |  | CKD3-5 | | |
| --- | --- | --- | --- | --- | --- | --- | --- | --- | --- | --- |
| ICD code | Diagnosis | No. of cases (IR/100 PY) |  | ICD code | Diagnosis | No. of cases (IR/100 PY) |  | ICD code | Diagnosis | No. of cases (IR/100 PY) |
|  |  |  |  |  | **A00‑B99 Infectious/Parasitic** |  |  |  |  |  |
| B34 | Viral infection of unspecified site | 704 (1.4) |  | A41 | Sepsis, unspecified organism | 255 (1.5) |  | A41 | Sepsis, unspecified organism | 1047 (2.2) |
| A46 | Erysipelas | 558 (1.1) |  | A46 | Erysipelas | 244 (1.5) |  | A46 | Erysipelas | 918 (2) |
| B37 | Candidiasis | 552 (1.1) |  | B34 | Viral infection of unspecified site | 222 (1.3) |  | B96 | Other bacterial agents as the cause of diseases classified elsewhere | 739 (1.6) |
| A41 | Sepsis, unspecified organism | 509 (1) |  | B96 | Other bacterial agents as the cause of diseases classified elsewhere | 203 (1.2) |  | B34 | Viral infection of unspecified site | 610 (1.3) |
| B02 | Zoster [herpes zoster] | 438 (0.9) |  | B37 | Candidiasis | 198 (1.2) |  | B37 | Candidiasis | 560 (1.2) |
|  |  |  |  |  | **C00‑D48 Neoplasms** |  |  |  |  |  |
| C44 | Other malignant neoplasms of skin | 655 (1.3) |  | C61 | Malignant neoplasm of prostate | 232 (1.4) |  | C44 | Other malignant neoplasms of skin | 1091 (2.3) |
| C61 | Malignant neoplasm of prostate | 545 (1.1) |  | C44 | Other malignant neoplasms of skin | 181 (1.1) |  | C61 | Malignant neoplasm of prostate | 780 (1.7) |
| D48 | Neoplasm of uncertain or unknown behaviour | 481 (0.9) |  | D48 | Neoplasm of uncertain or unknown behaviour | 152 (0.9) |  | D48 | Neoplasm of uncertain or unknown behaviour | 743 (1.6) |
| D37 | Neoplasm of uncertain or unknown behaviour of digestive organs | 325 (0.6) |  | D37 | Neoplasm of uncertain or unknown behaviour of digestive organs | 125 (0.8) |  | D37 | Neoplasm of uncertain or unknown behaviour of digestive organs | 413 (0.9) |
| C78 | Secondary malignant neoplasm of respiratory and digestive organs | 262 (0.5) |  | C67 | Malignant neoplasm of bladder | 121 (0.7) |  | C67 | Malignant neoplasm of bladder | 368 (0.8) |
|  |  |  |  |  | **D50‑D89 Blood/Immune** |  |  |  |  |  |
| D64 | Other anaemias | 1466 (2.9) |  | D64 | Other anaemias | 660 (4) |  | D64 | Other anaemias | 2741 (5.9) |
| D50 | Iron deficiency anaemia | 762 (1.5) |  | D50 | Iron deficiency anaemia | 335 (2) |  | D50 | Iron deficiency anaemia | 1059 (2.3) |
| D68 | Other coagulation defects | 174 (0.3) |  | D68 | Other coagulation defects | 68 (0.4) |  | D62 | Acute posthaemorrhagic anaemia | 284 (0.6) |
| D62 | Acute posthaemorrhagic anaemia | 159 (0.3) |  | D62 | Acute posthaemorrhagic anaemia | 64 (0.4) |  | D68 | Other coagulation defects | 280 (0.6) |
| D69 | Purpura and other haemorrhagic conditions | 114 (0.2) |  | D69 | Purpura and other haemorrhagic conditions | 42 (0.3) |  | D63 | Anaemia in chronic diseases classified elsewhere | 166 (0.4) |
|  |  |  |  |  | **E00‑E90 Endocrine/Metabolic** |  |  |  |  |  |
| E11 | Type 2 diabetes mellitus | 4245 (8.3) |  | E11 | Type 2 diabetes mellitus | 2031 (12.3) |  | E11 | Type 2 diabetes mellitus | 5181 (11.1) |
| E78 | Disorders of lipoprotein metabolism and other lipidaemias | 1449 (2.8) |  | E14 | Unspecified diabetes mellitus | 455 (2.7) |  | E87 | Other disorders of fluid, electrolyte and acid-base balance | 1118 (2.4) |
| E14 | Unspecified diabetes mellitus | 924 (1.8) |  | E10 | Type 1 diabetes mellitus | 388 (2.3) |  | E14 | Unspecified diabetes mellitus | 1016 (2.2) |
| E03 | Other hypothyroidism | 747 (1.5) |  | E78 | Disorders of lipoprotein metabolism and other lipidaemias | 365 (2.2) |  | E10 | Type 1 diabetes mellitus | 942 (2) |
| E10 | Type 1 diabetes mellitus | 710 (1.4) |  | E87 | Other disorders of fluid, electrolyte and acid-base balance | 299 (1.8) |  | E03 | Other hypothyroidism | 926 (2) |
|  |  |  |  |  | **F00‑F99 Mental/Behavioural** |  |  |  |  |  |
| F41 | Other anxiety disorders | 1136 (2.2) |  | F32 | Depressive episode | 333 (2) |  | F06 | Other mental disorders due to brain damage and dysfunction and to physical disease | 1092 (2.3) |
| F32 | Depressive episode | 967 (1.9) |  | F41 | Other anxiety disorders | 322 (1.9) |  | F41 | Other anxiety disorders | 948 (2) |
| F06 | Other mental disorders due to brain damage and dysfunction and to physical disease | 661 (1.3) |  | F06 | Other mental disorders due to brain damage and dysfunction and to physical disease | 294 (1.8) |  | F32 | Depressive episode | 913 (2) |
| F43 | Reaction to severe stress, and adjustment disorders | 472 (0.9) |  | F10 | Mental and behavioural disorders due to use of alcohol | 158 (1) |  | F03 | Unspecified dementia | 718 (1.5) |
| F00 | Dementia in Alzheimer's disease | 347 (0.7) |  | F03 | Unspecified dementia | 142 (0.9) |  | F00 | Dementia in Alzheimer's disease | 717 (1.5) |
|  |  |  |  |  | **G00‑G99 Nervous System** |  |  |  |  |  |
| G47 | Sleep disorders | 1313 (2.6) |  | G47 | Sleep disorders | 462 (2.8) |  | G47 | Sleep disorders | 1141 (2.4) |
| G45 | Transient cerebral ischaemic attacks and related syndromes | 426 (0.8) |  | G45 | Transient cerebral ischaemic attacks and related syndromes | 139 (0.8) |  | G30 | Alzheimer's disease | 713 (1.5) |
| G62 | Other polyneuropathies | 369 (0.7) |  | G30 | Alzheimer's disease | 138 (0.8) |  | G45 | Transient cerebral ischaemic attacks and related syndromes | 522 (1.1) |
| G30 | Alzheimer's disease | 340 (0.7) |  | G62 | Other polyneuropathies | 123 (0.7) |  | G62 | Other polyneuropathies | 378 (0.8) |
| G56 | Mononeuropathies of upper limb | 305 (0.6) |  | G40 | Epilepsy | 85 (0.5) |  | G56 | Mononeuropathies of upper limb | 226 (0.5) |
|  |  |  |  |  | **H00‑H59 Eye/Adnexa** |  |  |  |  |  |
| H25 | Age-related (senile) cataract | 2430 (4.7) |  | H25 | Age-related (senile) cataract | 802 (4.8) |  | H25 | Age-related (senile) cataract | 2300 (4.9) |
| H26 | Other cataract | 1150 (2.2) |  | H26 | Other cataract | 394 (2.4) |  | H35 | Other retinal disorders | 1532 (3.3) |
| H35 | Other retinal disorders | 978 (1.9) |  | H35 | Other retinal disorders | 305 (1.8) |  | H26 | Other cataract | 1238 (2.6) |
| H40 | Glaucoma | 753 (1.5) |  | H36 | Retinal disorders in diseases classified elsewhere | 304 (1.8) |  | H40 | Glaucoma | 1076 (2.3) |
| H10 | Conjunctivitis | 553 (1.1) |  | H40 | Glaucoma | 245 (1.5) |  | H36 | Retinal disorders in diseases classified elsewhere | 622 (1.3) |
|  |  |  |  |  | **H60‑H95 Ear/Mastoid** |  |  |  |  |  |
| H90 | Conductive and sensorineural hearing loss | 1868 (3.7) |  | H90 | Conductive and sensorineural hearing loss | 556 (3.4) |  | H90 | Conductive and sensorineural hearing loss | 2201 (4.7) |
| H61 | Other disorders of external ear | 1152 (2.3) |  | H61 | Other disorders of external ear | 418 (2.5) |  | H61 | Other disorders of external ear | 1670 (3.6) |
| H91 | Other hearing loss | 835 (1.6) |  | H91 | Other hearing loss | 248 (1.5) |  | H91 | Other hearing loss | 1057 (2.3) |
| H81 | Disorders of vestibular function | 516 (1) |  | H60 | Otitis externa | 171 (1) |  | H81 | Disorders of vestibular function | 565 (1.2) |
| H60 | Otitis externa | 503 (1) |  | H81 | Disorders of vestibular function | 162 (1) |  | H60 | Otitis externa | 389 (0.8) |
|  |  |  |  |  | **I00‑I99 Circulatory System** |  |  |  |  |  |
| **I10** | Essential (primary) hypertension | 6374 (12.5) |  | **I10** | Essential (primary) hypertension | 2325 (14) |  | I50 | Heart failure | 8578 (18.4) |
| I50 | Heart failure | 4501 (8.8) |  | I50 | Heart failure | 2190 (13.2) |  | **I10** | Essential (primary) hypertension | 7504 (16.1) |
| I25 | Chronic ischaemic heart disease | 3815 (7.5) |  | I48 | Atrial fibrillation and flutter | 1444 (8.7) |  | I48 | Atrial fibrillation and flutter | 5815 (12.4) |
| I48 | Atrial fibrillation and flutter | 3365 (6.6) |  | I25 | Chronic ischaemic heart disease | 1132 (6.8) |  | I25 | Chronic ischaemic heart disease | 2782 (6) |
| I21 | Acute myocardial infarction | 2601 (5.1) |  | I21 | Acute myocardial infarction | 804 (4.9) |  | I21 | Acute myocardial infarction | 2164 (4.6) |
|  |  |  |  |  | **J00‑J99 Respiratory System** |  |  |  |  |  |
| J06 | Acute upper respiratory infections of multiple and unspecified sites | 1841 (3.6) |  | J44 | Other chronic obstructive pulmonary disease | 746 (4.5) |  | J18 | Pneumonia, organism unspecified | 2428 (5.2) |
| J44 | Other chronic obstructive pulmonary disease | 1651 (3.2) |  | J18 | Pneumonia, organism unspecified | 629 (3.8) |  | J44 | Other chronic obstructive pulmonary disease | 1944 (4.2) |
| J18 | Pneumonia, organism unspecified | 1510 (3) |  | J06 | Acute upper respiratory infections of multiple and unspecified sites | 540 (3.3) |  | J15 | Bacterial pneumonia, not elsewhere classified | 1641 (3.5) |
| J15 | Bacterial pneumonia, not elsewhere classified | 892 (1.7) |  | J15 | Bacterial pneumonia, not elsewhere classified | 397 (2.4) |  | J06 | Acute upper respiratory infections of multiple and unspecified sites | 1522 (3.3) |
| J45 | Asthma | 797 (1.6) |  | J96 | Respiratory failure, not elsewhere classified | 274 (1.7) |  | J96 | Respiratory failure, not elsewhere classified | 853 (1.8) |
|  |  |  |  |  | **K00‑K93 Digestive System** |  |  |  |  |  |
| K59 | Other functional intestinal disorders | 1227 (2.4) |  | K59 | Other functional intestinal disorders | 439 (2.7) |  | K59 | Other functional intestinal disorders | 1688 (3.6) |
| K57 | Diverticular disease of intestine | 602 (1.2) |  | K92 | Other diseases of digestive system | 246 (1.5) |  | K92 | Other diseases of digestive system | 964 (2.1) |
| K92 | Other diseases of digestive system | 583 (1.1) |  | K57 | Diverticular disease of intestine | 171 (1) |  | K57 | Diverticular disease of intestine | 715 (1.5) |
| K21 | Gastroesophageal reflux disease | 502 (1) |  | K29 | Gastritis and duodenitis | 137 (0.8) |  | K29 | Gastritis and duodenitis | 411 (0.9) |
| K30 | Functional dyspepsia | 428 (0.8) |  | K21 | Gastroesophageal reflux disease | 126 (0.8) |  | K21 | Gastroesophageal reflux disease | 407 (0.9) |
|  |  |  |  |  | **L00‑L99 Skin/subcutaneous tissue** |  |  |  |  |  |
| L08 | Other local infections of skin and subcutaneous tissue | 1129 (2.2) |  | L98 | Other disorders of skin and subcutaneous tissue, not elsewhere classified | 501 (3) |  | L98 | Other disorders of skin and subcutaneous tissue, not elsewhere classified | 1710 (3.7) |
| L98 | Other disorders of skin and subcutaneous tissue, not elsewhere classified | 1081 (2.1) |  | L08 | Other local infections of skin and subcutaneous tissue | 475 (2.9) |  | L08 | Other local infections of skin and subcutaneous tissue | 1479 (3.2) |
| L82 | Seborrhoeic keratosis | 824 (1.6) |  | L97 | Ulcer of lower limb, not elsewhere classified | 319 (1.9) |  | L97 | Ulcer of lower limb, not elsewhere classified | 1230 (2.6) |
| L30 | Other dermatitis | 813 (1.6) |  | L30 | Other dermatitis | 244 (1.5) |  | L57 | Skin changes due to chronic exposure to non-ionizing radiation | 953 (2) |
| L57 | Skin changes due to chronic exposure to nonâ€‘ionizing radiation | 576 (1.1) |  | L82 | Seborrhoeic keratosis | 186 (1.1) |  | L30 | Other dermatitis | 846 (1.8) |
|  |  |  |  |  | **M00‑M99 Musculoskeletal System** |  |  |  |  |  |
| M79 | Other soft tissue disorders, not elsewhere classified | 3553 (6.9) |  | M79 | Other soft tissue disorders, not elsewhere classified | 1182 (7.1) |  | M79 | Other soft tissue disorders, not elsewhere classified | 3545 (7.6) |
| M54 | Dorsalgia | 2988 (5.8) |  | M54 | Dorsalgia | 915 (5.5) |  | M54 | Dorsalgia | 2757 (5.9) |
| M25 | Other joint disorders, not elsewhere classified | 2325 (4.5) |  | M25 | Other joint disorders, not elsewhere classified | 653 (3.9) |  | M25 | Other joint disorders, not elsewhere classified | 2087 (4.5) |
| M17 | Osteoarthritis of knee | 1008 (2) |  | M17 | Osteoarthritis of knee | 300 (1.8) |  | M10 | Gout | 1498 (3.2) |
| M48 | Other spondylopathies | 861 (1.7) |  | M10 | Gout | 281 (1.7) |  | M48 | Other spondylopathies | 1029 (2.2) |
|  |  |  |  |  | **N00‑N99 Genitourinary System** |  |  |  |  |  |
| N39 | Other disorders of urinary system | 1983 (3.9) |  | N39 | Other disorders of urinary system | 848 (5.1) |  | N18 | Chronic kidney disease | 5638 (12.1) |
| N30 | Cystitis | 1779 (3.5) |  | N30 | Cystitis | 678 (4.1) |  | N39 | Other disorders of urinary system | 3178 (6.8) |
| N40 | Hyperplasia of prostate | 874 (1.7) |  | N18 | Chronic kidney disease | 554 (3.3) |  | N30 | Cystitis | 2349 (5) |
| N18 | Chronic kidney disease | 677 (1.3) |  | N40 | Hyperplasia of prostate | 343 (2.1) |  | N17 | Acute kidney failure | 1584 (3.4) |
| N95 | Menopausal and other perimenopausal disorders | 641 (1.3) |  | N17 | Acute kidney failure | 311 (1.9) |  | N19 | Unspecified kidney failure | 1149 (2.5) |

## Table S7. Mortality and censoring rates in patients without any inpatient/outpatient events by CKD stage and follow-up duration

| CKD Stage | Follow-up | In-hospital N | In-hospital Death n (%) | In-hospital Censored n (%) | Outpatient N | Outpatient Death n (%) | Outpatient Censored n (%) |
| --- | --- | --- | --- | --- | --- | --- | --- |
| Overall | Follow-up <2.8 years | 4277 | 1461 (34.2%) | 2816 (65.8%) | 1526 | 1266 (83.0%) | 260 (17.0%) |
| Overall | Follow-up >=2.8 years | 1657 | 140 (8.4%) | 1517 (91.6%) | 23 | 20 (87.0%) | 3 (13.0%) |
| ckd0 | Follow-up >=2.8 years | 970 | 52 (5.4%) | 918 (94.6%) | 6 | 5 (83.3%) | 1 (16.7%) |
| ckd0 | Follow-up <2.8 years | 1611 | 347 (21.5%) | 1264 (78.5%) | 393 | 298 (75.8%) | 95 (24.2%) |
| ckd1-2 | Follow-up >=2.8 years | 210 | 11 (5.2%) | 199 (94.8%) | 2 | 1 (50.0%) | 1 (50.0%) |
| ckd1-2 | Follow-up <2.8 years | 612 | 214 (35.0%) | 398 (65.0%) | 201 | 171 (85.1%) | 30 (14.9%) |
| ckd3-5 | Follow-up >=2.8 years | 477 | 77 (16.1%) | 400 (83.9%) | 15 | 14 (93.3%) | 1 (6.7%) |
| ckd3-5 | Follow-up <2.8 years | 2054 | 900 (43.8%) | 1154 (56.2%) | 932 | 797 (85.5%) | 135 (14.5%) |

No- CKD

CKD1-2

CKD3-5

## Figure S2 Disease trajectories of primary hospitalization cause for survivors of a MI/HF stratified by the presence and severity of CKD in the region of Stockholm, Sweden, during 2006-2021. Disease trajectory panel (on the left) shows the most frequent chronological disease trajectories related to hospital admissions and ascertained by primary diagnoses codes according to ICD-10 chapter headings. Trajectories are ordered by frequency and only list those occurring in ≥0.5% of cases. The right column summarises the frequency and relative frequency in each trajectory across CKD groups.

No- CKD

CKD1-2

CKD3-5

## Figure S3 Disease trajectories of primary outpatent-specialist consultation cause for survivors of a MI/HF stratified by the presence and severity of CKD in the region of Stockholm, Sweden, during 2006-2021. Disease trajectory panel (on the left) shows the most frequent chronological disease trajectories related to hospital admissions and ascertained by primary diagnoses codes according to ICD-10 chapter headings. Trajectories are ordered by frequency and only list those occurring in ≥0.5% of cases. The right column summarises the frequency and relative frequency in each trajectory across CKD groups.

## STROBE Statement—Checklist of items that should be included in reports of cohort studies

|  | Item No | Recommendation | Page No |
| --- | --- | --- | --- |
| **Title and abstract** | 1 | (*a*) Indicate the study’s design with a commonly used term in the title or the abstract | 1,2 |
|  |  | (*b*) Provide in the abstract an informative and balanced summary of what was done and what was found |  |
| Introduction | | | |
| Background/rationale | 2 | Explain the scientific background and rationale for the investigation being reported | 3 |
| Objectives | 3 | State specific objectives, including any prespecified hypotheses | 3 |
| Methods | | | |
| Study design | 4 | Present key elements of study design early in the paper | 4 |
| Setting | 5 | Describe the setting, locations, and relevant dates, including periods of recruitment, exposure, follow-up, and data collection | 4 |
| Participants | 6 | (*a*) Give the eligibility criteria, and the sources and methods of selection of participants. Describe methods of follow-up | 4 |
|  |  | (*b*) For matched studies, give matching criteria and number of exposed and unexposed |  |
| Variables | 7 | Clearly define all outcomes, exposures, predictors, potential confounders, and effect modifiers. Give diagnostic criteria, if applicable | 4 |
| Data sources/ measurement | 8* | For each variable of interest, give sources of data and details of methods of assessment (measurement). Describe comparability of assessment methods if there is more than one group | 5 |
| Bias | 9 | Describe any efforts to address potential sources of bias | 5,6 |
| Study size | 10 | Explain how the study size was arrived at | 4 |
| Quantitative variables | 11 | Explain how quantitative variables were handled in the analyses. If applicable, describe which groupings were chosen and why | 4 |
| Statistical methods | 12 | (*a*) Describe all statistical methods, including those used to control for confounding | 5,6 |
|  |  | (*b*) Describe any methods used to examine subgroups and interactions |  |
|  |  | (*c*) Explain how missing data were addressed |  |
|  |  | (*d*) If applicable, explain how loss to follow-up was addressed |  |
|  |  | (*e*) Describe any sensitivity analyses |  |
| Results | | |  |
| Participants | 13* | (a) Report numbers of individuals at each stage of study—eg numbers potentially eligible, examined for eligibility, confirmed eligible, included in the study, completing follow-up, and analysed | 7 |
|  |  | (b) Give reasons for non-participation at each stage |  |
|  |  | (c) Consider use of a flow diagram |  |
| Descriptive data | 14* | (a) Give characteristics of study participants (eg demographic, clinical, social) and information on exposures and potential confounders | 7 |
|  |  | (b) Indicate number of participants with missing data for each variable of interest |  |
|  |  | (c) Summarise follow-up time (eg, average and total amount) |  |
| Outcome data | 15* | Report numbers of outcome events or summary measures over time | 8,9 |

| Main results | 16 | (*a*) Give unadjusted estimates and, if applicable, confounder-adjusted estimates and their precision (eg, 95% confidence interval). Make clear which confounders were adjusted for and why they were included | 8,9 |
| --- | --- | --- | --- |
|  |  | (*b*) Report category boundaries when continuous variables were categorized |  |
|  |  | (*c*) If relevant, consider translating estimates of relative risk into absolute risk for a meaningful time period |  |
| Other analyses | 17 | Report other analyses done—eg analyses of subgroups and interactions, and sensitivity analyses | NA |
| Discussion | | | |
| Key results | 18 | Summarise key results with reference to study objectives | 10 |
| Limitations | 19 | Discuss limitations of the study, taking into account sources of potential bias or imprecision. Discuss both direction and magnitude of any potential bias | 11,12 |
| Interpretation | 20 | Give a cautious overall interpretation of results considering objectives, limitations, multiplicity of analyses, results from similar studies, and other relevant evidence | 10-12 |
| Generalisability | 21 | Discuss the generalisability (external validity) of the study results | 12 |
| Other information | | | |
| Funding | 22 | Give the source of funding and the role of the funders for the present study and, if applicable, for the original study on which the present article is based | 12 |

*Give information separately for exposed and unexposed groups.

**Note:** An Explanation and Elaboration article discusses each checklist item and gives methodological background and published examples of transparent reporting. The STROBE checklist is best used in conjunction with this article (freely available on the Web sites of PLoS Medicine at http://www.plosmedicine.org/, Annals of Internal Medicine at http://www.annals.org/, and Epidemiology at http://www.epidem.com/). Information on the STROBE Initiative is available at http://www.strobe-statement.org.
